# Exposure and risk characterizations of ochratoxins A and aflatoxins through maize (*Zea mays*) consumed in different agro-ecological zones of Ghana

**DOI:** 10.1038/s41598-021-02822-x

**Published:** 2021-12-02

**Authors:** Nii Korley Kortei, Theophilus Annan, Vincent Kyei-Baffour, Edward Ken Essuman, Harry Okyere, Clement Okraku Tettey

**Affiliations:** 1 Department of Nutrition and Dietetics, School of Allied Health Sciences, University of Health and Allied Sciences, PMB 31, Ho, Ghana; 2 Food Microbiology Division, Council for Scientific and Industrial Research- Food Research Institute, P. O. Box M20, Accra, Ghana; 3 Food Chemistry and Nutrition Research Division, Council for Scientific and Industrial Research- Food Research Institute, P. O. Box M20, Accra, Ghana; 4 Council for Scientific and Industrial Research- Crops Research Institute, P. O. Box 3785, Fumesua, Kumasi, Ghana; 5 Department of Biomedical Sciences, School of Basic and Biomedical Sciences, University of Health and Allied Sciences, PMB 31, Ho, Ghana

**Keywords:** Microbiology, Environmental sciences, Natural hazards, Pathogenesis

## Abstract

Mycotoxin contamination of foodstuffs is a serious food safety concern globally as the prolonged ingestion of these toxins has the tendency to worsen the risk of hepatocellular carcinoma. This study aimed at estimating ochratoxin A (OTA) and aflatoxin (AF) levels above international (European Food Safety Authority, EFSA) and local (Ghana Standards Authority, GSA) standards as well as the health risks associated with the consumption of maize (n = 180) sampled from six (6) regions representing the agro-ecological zones of Ghana. OTA and AF were measured with High-Performance Liquid Chromatography with a Fluorescence detector. Out of the 180 samples analyzed for total aflatoxins (AFtotal), 131/180 tested positive and 127 (70.50%) exceeded the limits of EFSA and ranged 4.27–441.02 µg/kg. While for GSA, 116 (64.44%) of samples exceeded this limit and ranged between 10.18 and 441.02 µg/kg. For OTA, 103/180 tested positive and 94 (52.22%) of samples between the range 4.00–97.51 µg/kg exceeded the tolerable limit of EFSA, whereas 89 (49.44%) and were in the range of 3.30–97.51 µg/kg exceeded the limits of GSA. Risk assessment values for total aflatoxins (AFtotal) ranged between 50 and 1150 ng/kg bw/day, 0.4–6.67, 0–0.0323 aflatoxins ng/kg bw/day and 1.62–37.15 cases/100,000 person/year for Estimated Daily Intake (EDI), Margin of Exposure (MOE), Average Potency, and Cancer Risks respectively. Likewise, ochratoxin (OTA) values were in the ranges of 8.6 × 10^–3^–450 ng/kg bw/day, 0.05–2059.97, 0–0.0323 ochratoxins ng/kg bw/day and 2.78 × 10^–4^–14.54 cases/100,000 person/year. Consumption of maize posed adverse health effects in all age categories of the locations studied since the calculated MOE values were less than 10,000.

## Introduction

Maize (*Zea mays* L), a principal cereal extensively consumed^1^ around the globe is exceedingly prone to fungal infection by many toxigenic fungal species. This subsequently leads to mycotoxins (fungal toxins) production^2,3^ due to its ideal nutrient composition^4^. This situation is particularly distressing since maize (*Zea mays*) accounts for 40% of the cereal production in Sub-Saharan Africa (SSA), where more than 80% is used as food^5^. The crop provides at least 30% of the total calorie intake of people in Sub-Saharan Africa. Maize is consumed as a staple in the African region where intake ranges from 52 to 450 g/person/day^1,6^ and so is an easy channel for mycotoxin contamination. Mycotoxins, which are natural toxins from fungi, contaminate maize grains and render them potentially dangerous. Mycotoxins represent one of the main global foodborne risks for human health, and are considered an important issue in the situation of food safety, due to their acute and chronic toxic effects on animals and humans^7,8^. Noteworthy, mycotoxins of maize include aflatoxins (AFs), ochratoxin A (OTA), and Fumonisins (FM)^9–11^. Attendant contamination could either be single or occur in multiple combinations with other mycotoxins. Ochratoxins are fungal metabolites produced from the growth of some notable species including *Aspergillus ochraceus*, *A. carbonarius, A. niger,* and *Penicillium verrucosum*. Ochratoxins occur in A, B, C, and D types, of which the most common and toxic type is Ochratoxin A (OTA). OTA is assumed to be one of the five most important mycotoxins in agriculture^12^. Although not well known in Africa and the world at large compared to aflatoxins, ochratoxins are described as one of the most commonly occurring, mycotoxins in the world due to their presence in a wide variety of foodstuffs such as potatoes, pulses, nuts, spices coffee, cacao, beer, and wine^10^. Nonetheless, notable among the main foodstuffs are cereals and cereal products, which constitute 60% of its total exposure to man and animals according to^13^ (JECFA).

The toxicological profile of OTA has been investigated and reviewed expansively in numerous studies^14–19^. In summary, these studies showed that OTA is nephrotoxic, hepatotoxic, neurotoxic, teratogenic, mutatoxic, immunotoxic and causes blood–brain barrier damage in various animals and humans, with renal toxicity and carcinogenesis being significant adverse effects. There is limited evidence of OTA-associated chronic kidney disease in humans^12^. Ochratoxins A (OTA) is aptly classified as a possible human carcinogen (2B agent) according to the International Agency for Research on Cancer (IARC)^12,20^. Aflatoxins are also fungal metabolites produced by strains of *Aspergillus flavus and A. parasiticus*. They are by and large the most well-known mycotoxins owing to their persistence in the environment and the ubiquitous nature of their contaminants. Aflatoxins occur in five different types; aflatoxins B_1_, B_2_, G_1_, G_2_, and M_1_ produced primarily in cow milk by cow-eating contaminated silage. Prolonged ingestion of aflatoxins has been reported to cause impaired immune function, hepatotoxicity, neurotoxicity, malnutrition, and stunted growth in children, teratogenic, mutagenic disabilities, and eventual death^21,22^. The International Agency for Research on Cancer (IARC) and the Joint FAO/WHO Expert Committee on Food Additives (JECFA) named it as a class 1 carcinogen due to its high potency^23,24^.

The evidence that mycotoxins can have adverse health effects on humans and animals has led many countries to set up standards for maximum levels of total aflatoxins and ochratoxins in food products intended for both human and animal consumption^25^. These permissible levels are almost excruciating especially in developing countries in Sub-Saharan Africa. Bankole and Adebanjo^26^ noted when the worldwide directive of mycotoxins in food is under consideration, the impact seems to be greater on low to medium-income countries. It was projected that enforcement of strict regulations regarding aflatoxin contamination by the European Union would result in the rejection of 64% of imports of cereals, dried fruits, and nuts from African countries with an estimated trade loss per year of approximately US $670 million^26^. In addition to setting regulatory limits for mycotoxins, it is also imperative to conduct health risk assessments in the population due to dietary exposure. A low-dose extrapolation approach introduced by the Joint FAO/WHO Expert Committee on Food Additives (JECFA) in 1997 and the margin of exposure (MOE) method proposed at the 64th JECFA meeting in 2005^27^ were both recommended and have been widely used worldwide^28,29^ to assess the risk of dietary exposure to mycotoxins.

There is a paucity of data on the combined occurrence of ochratoxin A and aflatoxin contamination in foodstuffs in Ghana. Previous works by national institutions such as the Food and Drugs Authority (FDA), Ghana, Ghana Standards Authority (GSA), and Council for Scientific and Industrial Research (CSIR), Ghana have conducted surveillance programmes to monitor contamination by only aflatoxins in foodstuffs. To the best of our knowledge, not a single work is published on the exposure assessment of the co-occurrence of OTA and AFs as well as their risk characterization. This study is the only study in Ghana that assessed the levels, exposure and risk characterization of ochratoxin A and aflatoxins through the consumption of maize in different age group samples from six (6) different regions representative of the different agro-ecological zones of Ghana by using MOE and quantitative liver cancer risk approaches.

## Materials and methods

### Sample collection

To collect a representative data set, we first obtained the list of villages in each district from the Regional Directorate of the Ministry of Agriculture. From each district, an average of 5 villages (Table 1) was then randomly selected. The maize sellers in each market were conveniently sampled where about one kilogram (1 kg) of raw maize samples were purchased concurrently from July to December 2020. Five hundred (500) grams each of maize samples were fetched and kept in sterile bags in ice chests and sent to the laboratory within the same day in a vehicle where they were stored in a deep freezer at − 20 °C until ready for chemical analysis^30^.

**Table 1:** Geographical locations and some attributes of the origin of samples.

Region	No. of samples	Agro-ecological zone	Rainfall (mm)	Temperature (°C)	Coordinates
Upper East	30/180	Sudan Savannah	800–1000	28.3	10.7082 °N, 0.9821 °W
Northern	30/180	Guinea Savannah	1000–1200	27.9	9.5439 °N, 0.9057 °W
Ashanti	30/180	Transitional	1200–1400	25.9	6.7470 °N, 1.5209 °W
Eastern	30/180	Semi-deciduous	1400–1900	25.9	6.2374 °N, 0.4502 °W
Central	30/180	Coastal Savannah	1400–1600	26.7	5.5608 °N, 1.0586 °W
Western	30/180	Rain Forest	1800–2000	25.9	5.3902 °N, 2.1450 °W

### Determination of Ochratoxins A

#### Chemicals and standards

The analytical standard of OTA was supplied by Sigma-Aldrich (St. Louis, MO, USA). All solvents used for the preparation of the mobile phase were HPLC grade and obtained from Merck (Darmstadt, Germany). Methanol and hexane used for extraction were of analytical grade supplied by Sigma-Aldrich. All homogenized mixtures and eluates were filtered through Whatman no. 4 and 0.45 mm membrane filters respectively (Whatman plc, Maidstone, UK). De-ionized water was obtained with a Millipore Elix Essential purification system (Bedford, MA, USA). OCHRA PREP immunoaffinity columns were supplied by R-Biopharm, Rhone limited, and used for SPE and cleanup. These columns have a concentration capacity of 100 ng/mL with at least 90% recovery. Phosphate-buffered saline (PBS) was prepared by dissolving PBS tablets (Sigma-Aldrich) in distilled water. Sodium chloride (≥ 99.0%) was sourced from Sigma-Aldrich. Six-point calibration was made using the pure Ochratoxin A standard at concentrations of 5 µg/kg, 10 µg/kg, 15 µg/kg, 20 µg/kg, 25 µg/kg and 30 µg/kg. Linearity was accepted at 0.99 or 99% for the regression curve (CEN official method EN14123^33^).

#### Determination of Ochratoxin

Ochratoxin A was determined based on CEN official method EN14123 (2007)^32^. About 500 g each of maize was sampled by thoroughly mixing and heaping the whole batch into a cone. Using cardboard, the heap was divided into four equal parts. Two opposite parts were mixed, and the remaining two parts were packed, and the process repeated until a representative 500 g sample was achieved and ground into fine maize powder and groundnut slurry. Exactly 25 g of powdered or slurred samples were extracted with 5 g Sodium Chloride and 200 mL methanol in distilled water in a ratio of 4:1, respectively. Hexane (100 mL) was added to the groundnut mixture and the samples homogenized for 3 min (i.e., 3000 rpm for 2 min and at 3500 rpm for 1 min). The groundnut mixture generated two organic layers (the hexane upper layer and methanol lower layer). The lower methanol layer of the groundnut mixture and the maize mixture was filtered through Whatman number 4 filter paper. Ten milliliters (10 mL) of filtrate were used for Ochratoxin A solid-phase extraction and cleanup. Exactly 150 mL of phosphate buffer saline (PBS) was added to 10 mL of filtrate and the mixture stirred. Immuno-affinity columns specific for Ochratoxin A were pre-conditioned and antibodies in the column were activated by eluting 10 mL of phosphate buffer saline through columns at a flow speed of 3 mL/min. Exactly 50 mL of the 160 mL filtrate-PBS mixture was loaded onto pre-conditioned immune-affinity columns specific for Ochratoxin A and allowed to drain by gravity. The columns were washed three times with 5 mL PBS and allowed to elute at a flow rate of 5 mL/min. Using a vacuum pump, the air was blown through the columns to get rid of all wash solvent molecules. Ochratoxin A was eluted in two steps into a 5 mL volumetric flask by first eluting with 1 mL of methanol (highest grade) followed by another 1 mL of methanol after one minute. Air was blown through the column to collect all eluates. Aqueous acetic acid (1%) was used to make up the volume of eluate to 4 mL and eluate vortexed, after which 2 mL was pipetted into HPLC vials for quantification.

#### HPLC parameters

Agilent high-performance liquid chromatography system (HPLC 1260 infinity series) with a quaternary pump and fluorescence detection was used for OTA quantification. Data acquisition and quantification were done using Chem station (Open Lab edition). The Agilent HPLC equipped with a fluorescence detector was set at an excitation wavelength of 333 nm and an emission wavelength of 467 nm and the column compartment temperature regulated at 30 °C. The mobile phase was a mixture of 5 mM sodium acetate with acetic acid (pH 2.4): methanol: acetonitrile at ratios of 40:30:30, respectively, and an isocratic delivery mode employed at a flow rate of 1 mL/min with an injection volume of 10 µl. The run time was set at 10 min (CEN official method EN14123^33^).

### Aflatoxins determination

#### Extraction of samples

AFB_1_, AFB_2_, AFG_1_, and AFG_2_ were extracted from the samples according to the European Committee for Standardization (CEN) official method EN14123^33^ for aflatoxin extraction. Methanol in water (200 mL) (8 + 2) and 5 g NaCl were used to extract 25 g of sample. Hexane (100 mL) was added to samples containing more than 50% fat. The mixture was homogenized for 3 min at 3000 rpm (2 min) and 3500 rpm (1 min). The extracts were filtered and 10 mL of the filtrate added to 60 mL of phosphate buffer saline (PBS) for solid-phase extraction using a preconditioned immune-affinity column specific for, AFB_1_, AFB_2_, AFG_1_, and AFG_2_. The 70 mL filtrate-PBS mixture was loaded onto the preconditioned column and allowed to elute by gravity at a flow rate of 1 mL/min. This was followed by a cleanup with 15 mL distilled water at a flow rate of 5 mL/min. Aflatoxins were eluted in two steps into a 5 mL volumetric flask with 0.5 mL followed by 0.75 mL of methanol (HPLC grade) and allowed to elute by gravity. Deionized water was used to make up the volume of eluate to 5 mL and eluate vortexed and 2 mL pipetted into HPLC vials for quantification.

#### HPLC parameters

Injection volume: 10 μl flow rate: 1 mL/min, column temperature: 35 ℃, excitation wavelength: 360 nm, emission wavelength: 440 nm, mobile phase composition: water/acetonitrile/MeOH (65:15:20 v/v/v), post-column derivatization: Kobra cells. HPLC Column Specification Spherisorb ODS1- Excel

- (4.6 mm × 25 cm), 5 μm particle size, 250 A pore size
- LOD = Limit of detection
- LOQ = Limit of quantification
- ACN = Acetonitrile
- MeOH = Methanol
- LOD calculation = 3 * standard deviation/slope
- LOQ calculation = 3 × LOD
- Supplier of Column R- Biopharm, Block 10 campus, West Scotland
- Science Park, Acre Road, Glasgow, Scotland G20 OXA

#### Analysis of samples

The aflatoxins (by *Aspergillus flavus* and *A. parasiticus*) levels in the samples were determined according to the CEN official method EN14123^33^ by High-Performance Liquid Chromatography HPLC (Agilent 1260 Series, OpenLab software, X-bridge column) (250 mm × 4.6 mm, i.d., 5 μm), USA with fluorescence detector and post-column derivatization using Kobra cells to generate bromine electrochemically at the CSIR- Food Research Institute, Ghana.

LOD for Ochratoxins was 0.83 µg/kg while LOQ recorded 2.49 µg/kg (Table 2).

**Table 2:** Limits of Detection (LOD) and Quantification (LOQ) of ochratoxins (OTA) and aflatoxins (AFB_1_, AFB_2_, AFG_1_, AFG_2_, and AFtotal) (µg/kg) measured by HPLC.

Mycotoxin	Limits	Amount (µg/kg)
OTA	LOD	0.83
	LOQ	2.49
AFB1	LOD	0.15
	LOQ	0.45
AFB2	LOD	0.15
	LOQ	0.45
AFG1	LOD	0.13
	LOQ	0.39
AFG2	LOD	0.13
	LOQ	0.39

LOD-Limit of Detection.

LOQ-Limit of Quantification.

LOD for AFB_1_, AFB_2_ was 0.15 µg/kg, and 0.13 µg/kg for AFG_1_ and AFG_2_, while LOQ were 0.39 and 0.45 µg/kg (Table 2).

#### Limit of detection/quantification (LOD/LOQ)

Limits of detection and quantification (LOD/LOQ) of the HPLC were estimated by making a calibration curve around the standard used for spiking, 5 µ/kg (the lowest concentration range of the calibration curve). Blank did not produce any signal, so the LOD and LOQ were calculated as;

1 $$ {\text{LOD}} = {3}*{\text{standard}}\,{\text{deviation}}/{\text{slope}}. $$

2 $$ {\text{LOQ}} = {3}*{\text{LOD}}. $$

#### Measurement accuracy

Spiking of pure aflatoxin standard solution was done to ensure the measurement accuracy of the analysis. Three levels of spiking were done at the lower, mid, and upper concentration range of the calibration curve concentrations (5 ppb, 15 ppb, and 30 ppb). Spike volumes of pure standards were calculated as;

3 $$ \left[ {{\text{Sample}}\,{\text{weight }}\left( {\text{g}} \right)*{\text{spike}}\,{\text{concentration}}\,\left( {{\text{ppb}}} \right)} \right]/\left[ {{\text{Concentration}}\,{\text{of}}\,{\text{standard}}\,\left( {{\text{ug}}/{\text{mL}}} \right)} \right]. $$

Spike volumes were distributed evenly on aflatoxin free sample (blank) and the spiked sample analyzed for percentage recovery which was calculated as;

4 $$ [({\text{Concentration}}\,{\text{measured}}\,{\text{in}}\,{\text{spike}}{-}{\text{Concentration}}\,{\text{measured}}\,{\text{in}}\,{\text{the}}\,{\text{blank}})/({\text{spiked}}\,{\text{amount}})]*{1}00 $$

#### Measurement precision

Repeatability and intermediate precision analyses of an internal reference material (IRM) were used to ensure the measurement precision of the method. For repeatability analysis, 10 parallel extractions of the IRM were done by the same analyst at the same time using the same HPLC and the relative standard deviation between the results was calculated. For intermediate precision, 10 extractions of the IRM were done on different days by different analysts, and the relative standard deviation between the results was calculated. The relative standard deviations were calculated as; [Standard deviation/mean] * 100 (CEN official method EN14123^33^).

#### Required performance criteria for accuracy and precision

Repeatability: Relative standard deviation among repeatable results should be less than 15%**.**

Intermediate Precision: Relative standard deviation among results obtained under intermediate precision conditions should be less than 20%**.**

- Recovery: Percent recovery of the measurement procedure should be in the range of 80–120%.
- Limit of Detection: The limit of detection should be less than 1 ug/kg for all aflatoxins.
- Limit of Quantification**:** The limit of quantification should be less than 3 ug/kg for all aflatoxins.
- Linearity**:** Linearity of the regression curve should be 0.99 (B1, B2, G1) and 0.98 (G2).

#### Experimental data

Repeatability: Relative standard deviation;

B1 = 5.5%; B2 = 6.7%; G1 = 7.4%; G2 = 12.1% and Total aflatoxins = 5.2%.

Intermediate Precision (Reproducibility): Relative standard deviation;

B1 = 13.2%; B2 = 13.4%; G1 = 13.7%; G2 = 12.2% and Total aflatoxins = 11.9%.

Recovery**:** Percent recovery of measurement procedure;

Low concentration: B1 = 107%; B2 = 87.2%; G1 = 113.4%; G2 = 112.8% and Total aflatoxins = 108.2%.

High concentration: B1 = 102.6%; B2 = 101.6%; G1 = 104.2%; G2 = 104.4% and Total aflatoxins = 103.3%.

Linearity: Linearity of the regression curve;

B1 = 0.991; B2 = 0.997, G1 = 0.994; G2 = 0.995 (CEN official method EN14123^33^).

### Risk assessment of exposure to total aflatoxins via consumption of maize

#### Exposure estimation

Estimated Daily Intake (EDI) was considered by using the mean quantities of mycotoxins (ochratoxins or aflatoxins) derived from the cereal samples, the number of samples consumed daily, and the average body weight. The EDI for mean aflatoxin was premeditated according to the following formula (5) and expressed in μg/kg of body weight/day (μg/kg bw/day)^34,35^.

5 $$ {\text{EDI}} = \frac{{daily{\text{int}}ake\left( {{\text{food}}} \right) \times {\text{mean level of mycotoxin}}}}{{\text{average bodyweight}}} $$

The daily intake of maize in Ghana according to MOFA-IFPRI (2020) in Kortei et al.^2^ is approximately 0.107 kg/day (39.3 kg/year).

The different age categories according to EFSA^36^ and their corresponding estimated average weights in Ghana used in this study were done as follows; Infants- 2.9 (2.5–3.2) kg^37,38^, Toddler- 9.8 (7–12.6) kg^39,40^ , Children- 26 (24–28) kg^41,42^, Adolescents- 46.25 (38.5–54) kg^43^ , Adults- 60.7 kg^44^.

#### Margin of exposure characterization for aflatoxins and ochratoxins

Genotoxic compounds such as aflatoxins and ochratoxins have their risk assessments fittingly computed based on the Margin of Exposure (MOEs) approach, which was estimated by dividing the Benchmark dose lower limit (BMDL) for aflatoxins is 400 ng/kg bw/day by toxin exposure^23,45^.

Similarly, Benchmark dose lower limit (BMDL) for ochratoxins—130 ng/kg bw/week^46^ and 120 ng/kg bw/week^47^ resulting in an average of 125 ng/kg bw/week (17.86 ng/kg bw/day) by toxin exposure as expressed in Eq. (6).

6 $$ {\text{MOE}} = \frac{{\text{Benchmark dose lower limit}}}{{{\text{EDI }}\left( {{\text{Exposure}}} \right)}} $$

A public health alarm is raised in instances where MOEs are less than 10,000 for both ochratoxin A and aflatoxins.

#### Estimated liver cancer risk due to consumption of maize

The ingestion of aflatoxins, likewise ochratoxins, can be linked to the onset of liver cancer^48,49^. Therefore, liver cancer risk estimation for Ghanaian adult consumers was calculated for aflatoxins^45^. This involved estimating the population cancer risk per 100,000, which is a product of the EDI value and the average hepatocellular carcinoma (HCC) potency figure from individual potencies of Hepatitis B surface antigen (HBsAg) (HBsAg-positive and HBsAg-negative groups).

The JECFA^50^ estimated potency values for AF/OTA which corresponded to 0.3 cancers/year/ 100,000 population/ng/kg bw/day (uncertainty range: 0.05–0.5) in HBsAg-positive individuals and 0.01 cancers/year/100,000 population/ng/kg bw/day (uncertainty range: 0.002–0.03) in HBsAg-negative individuals^49^ were adopted for this calculation. Moreover, the average HBsAg + prevalence rate of 7.74% (adult-8.36%, adolescents-14.3%, children-0.55%) for Ghana^51,52^ was adopted and 92.26% (100–7.74%) was extrapolated for HBsAg-negative groups. Hence, the average potency for cancer in Ghana was estimated as follows according to Eq. (7) as prescribed by^49^ and^45^.

7 $$ \begin{aligned} {\text{Average potency}} & = \left[ {0.0{3} \times {\text{HBsAg}}{-}{\text{negative individuals in Ghana}}} \right] \\ & \quad + \left[ {0.0{1} \times {\text{HBsAg}} - {\text{ positive individuals}}/{\text{prevalence rate in Ghana}}} \right] \\ \end{aligned} $$

$$ \begin{aligned} & \left( {0.{3 } \times \, 0.0{77}} \right) \, + \, \left( {0.0{1 } \times \, 0.{9226}} \right) \\ & \quad = 0.0{323} \\ \end{aligned} $$

Thus, the cancer risk (cancers per year per 100,000 population per ng aflatoxins/ochratoxins /kg bw/day) was estimated using the following formula in Eq. (8)^2,45^:

8 $$ {\text{Cancer risk}} = {\text{Exposure}}\,\left( {{\text{EDI}}} \right)\, \times \,{\text{Average potency}} $$

The use of plants in the present study complies with international, national, and/or institutional guidelines.

### Statistical analysis

The ochratoxins and aflatoxin concentrations were calculated using regression analysis from the curves generated from the standards of ochratoxins/aflatoxins with Excel for Microsoft Windows (version 10). SPSS 22 (Chicago, USA) was used in the analysis of data. Descriptive analysis was performed to describe the concentration of ochratoxins/aflatoxins in maize samples by using the mean ± standard deviation. Deterministic risk assessment model calculations for ochratoxins and aflatoxins dietary exposure (Estimated Dietary Intake), MOE values, Average potency, and cancer risk were calculated. The results are summarized as standard error, median, standard deviation, variance, skewness, standard error of skewness, kurtosis, and standard error of kurtosis and mean values (range from the 25th percentile to the 75th percentile). Associations of mycotoxins (AFtotal, AFB_1_, and Ochratoxins) were determined using Kendall’s tau_b correlation test.

## Results

### Occurrence of aflatoxins and ochratoxins A

**Figure 1:**
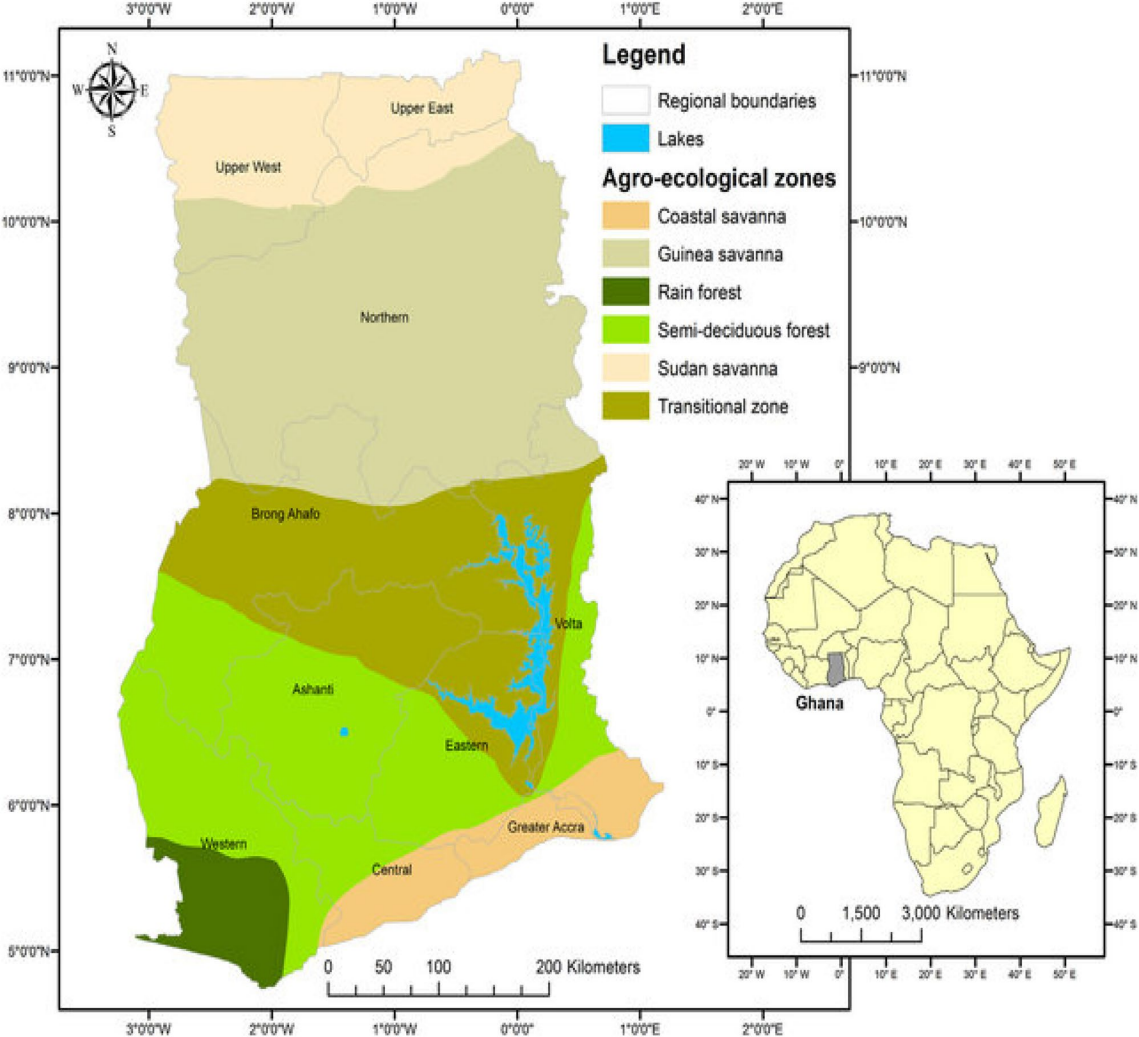
Map of Ghana showing the different agro-ecological zones where the maize grains were sampled (map adapted from^31^).

Samples tested produced good linearity or coefficients of correlations (R^2^ > 0.990) within the tested range. For the recovery analysis, samples previously tested to guarantee the nonappearance of the studied mycotoxins were used in the validation procedure. For ochratoxins, the limit of detection was 0.83 µg/kg while the Limits of Detection for AFB_1_ and AFB_2_, likewise AFG_1_ and AFG_2_, ranged between 0.13 and 0.15 µg/kg. The limit of Quantification for ochratoxins was 2.49 µg/kg. Aflatoxins ranged between 0.39 and 0.45 µg/kg, respectively, for both (Table 2). Out of a total of one hundred and eighty (180) samples tested, 131 tested positive. The general trend of occurrence of aflatoxins was in the decreasing order of AFB_1_ > AFB_2_ > AFG_1_ > AFG_2_ and were in the ranges of 0–337 µg/kg, 0–101.00 µg/kg, 0–24.80 µg/kg, and 0–5.51 µg/kg respectively. The aggregated aflatoxins (AFtotal) were in the range of 0–441.02 µg/kg. While for ochratoxins (OTA), 103 samples tested positive. OTA levels were also observed to be lesser than AFB_1_, AFB_2_ but more than AFG_1_ and AFG_2_ and ranged between 0 and 97.51 µg/kg. There were significant (*p* < 0.05) differences observed in all categories of the tested samples. For the Upper East region representing the Sudan Savanna zone (Fig. 1), the range of values was 0–106.18 µg/kg for Total aflatoxins. 32.84, 30.35, and 668.51 µg/kg were recorded from the summary statistics as mean, median, and variance, respectively, while 0.83 and 0.89 were recorded as skewness and kurtosis respectively which implied a symmetrical and normally distributed data for Total Aflatoxins (AFtotal) (the distribution is not outside the range of normality) (Table 3). For ochratoxins (OTA), values of the range 0–62.50 µg/kg were recorded. While 18.22, 14.56, and 371.75 represented the mean, median, and variance. Skewness and kurtosis were 0.77 and − 0.50, respectively, and the implied distribution produced fewer and less extreme outliers than did the normal distribution or were fairly symmetrical and light-tailed. There was a significant (*p* < 0.01) correlation between AFB_1_ and AFtotal in this data set (Table 4). The Northern Region (Guinea Savanna) zone recorded a range of values of 0–285.31 for Total aflatoxins. Values of 48.93, 25.09, and 4214.84 µg/kg were recorded for mean, median, and variance, respectively, while the skewness and kurtosis were 2.14 and 5.35 respectively and showed that the data set of Total aflatoxins (AFtotal) obtained in this zone was asymmetrical and heavy-tailed (Table 5). Ochratoxins (OTA), were within the range of 0–46.77 µg/kg. Values of 14.14, 6.70, and 232.47 µg/kg were recorded as mean, median, and variance, respectively, while 0.85 and − 0.58 were obtained as skewness and kurtosis respectively which suggested a fairly symmetrical distribution and light-tailed. There were significant (*p* < 0.01) correlations established between AFB_1_ and AFtotal as well as AFtotal and OTA (Table 6). Total aflatoxins for the Ashanti region representing Transitional zones were within the range of 0–230.00 µg/kg, while the mean, median, and variance recorded were 62.37, 46.77 and 4247.89 µg/kg respectively. The data set showed symmetrical and light-tailed as the skewness and kurtosis were 1.12 and 0.71, respectively (Table 7). Ochratoxins were also in the range of 0–76.05 µg/kg. Mean, median, and variance were 11.52, 0.00, and 403.94 µg/kg, respectively, with Skewness and Kurtosis of 1.70 and 2.5, suggesting the distribution is not outside the range of normality (Table 7). Significant (*p* < 0.01) correlations were established between AFB_1_ and AFtotal, and likewise AFtotal and OTA (Table 8). For Eastern Region representing the Semi-deciduous zones recorded a range of 0–441.02 µg/kg. Mean, median, and variance of 53.11, 4.83, and 10,840.01 µg/kg, respectively, were recorded. The data set for the Semi-deciduous zone was asymmetrical and light-tailed (2.48 and 6.24 for skewness and kurtosis, respectively, for Total aflatoxins (AFtotal) (Table 9). Ochratoxins levels ranged between 0 and 97.51 µg/kg. Mean, median, and variance recorded were 17.75, 4.10, and 827.54 µg/kg, respectively. The data set was symmetrical and light-tailed (Skewness and Kurtosis were 1.78 and 1.97 respectively). Significant (*p* < 0.01) correlations were recorded between AFB_1_ and AFtotal, and likewise AFtotal and OTA (Table 10). The Central Region representing the Coastal Savanna zone recorded a range of 0–139.71 µg/kg. Values of 30.68, 20.79, and 1418.09 µg/kg were recorded as the mean, median, and variance of the dataset. Skewness and Kurtosis were 1.54 and 1.65, respectively, which suggested a normal distribution and light-tailed (Table 11). For ochratoxins, a range of 0–38.34 µg/kg was recorded. Mean, median, and variance of 4.92, 0.00, and 80.94 µg/kg with skewness and kurtosis of 0.43 and 6.07 suggestive of a fairly symmetrical and heavy-tailed data set. Total aflatoxins (AFtotal) were significantly (*p* < 0.01) correlated with AFB_1_ and OTA (Table 12). Lastly, the Western Region representing the Rain Forest zone of Ghana ranged between 0 and 217.41 µg/kg. Values of 62.12, 48.64, and 3553.22 µg/kg were recorded as mean, median, and variance, respectively. Dataset was asymmetrical and light-tailed (skewness and kurtosis were 1.16 and 0.99) (Table 13). Quantities of ochratoxins were in the range of 0–85.90 µg/kg with mean, median, and variance of 24.19, 21.75, and 591.44 µg/kg respectively. The data was symmetrical and light-tailed (skewness and kurtosis were 0.93 and 0.19, respectively. Associations of Total aflatoxins (AFtotal) with AFB_1_ and OTA were found to be significant (*p* < 0.01) (Table 14).

**Table 3:** Statistical summary of data obtained from the Upper East Region of Ghana.

N samples (30)	AFB_1_	AFB_2_	AFG_1_	AFG_2_	AFtotal	OTA
Mean	26.47	4.54	1.47	0.37	32.84	18.22
Std. Error of Mean	4.05	0.69	0.32	0.14	4.72	3.52
Median	23.89	4.67	0.71	0.00	30.35	14.56
Std. Deviation	22.20	3.79	1.74	0.77	25.86	19.28
Variance	492.85	14.39	3.02	0.59	668.51	371.75
Skewness	1.01	0.59	0.96	1.81	0.83	0.77
Std. Error of Skewness	0.43	0.43	0.43	0.43	0.43	0.43
Kurtosis	1.60	− 0.41	0.35	1.76	0.89	− 0.50
Std. Error of Kurtosis	0.83	0.83	0.83	0.83	0.83	0.83
t	6.53	6.56	4.63	2.61	6.96	5.18
Sig. (2-tailed)	0.000	0.000	0.000	0.014	0.000	0.000
Minimum	0.00	0.00	0.00	0.00	0.00	0.00
Maximum	95.29	14.20	6.30	2.50	106.18	62.50
Percentiles 25	6.82	0.00	0.00	0.00	10.50	0.00
50	23.89	4.67	0.71	0.00	30.25	14.56
75	40.27	6.50	2.73	0.00	50.17	34.41

**Table 4:** Associations of mycotoxins (AFB_1_, AFtotal and OTA) in maize from Upper East Region.

	AFB_1_	AFtotal	OTA
AFB_1_	1.00	0.925**	0.162
		0.00	0.228
AFtotal		1.00	0.172
			0.201
OTA			1.000

**Correlation is significant at the 0.01 level (2-tailed).

**Table 5:** Statistical summary of data obtained from the Northern Region of Ghana.

N samples (30)	AFB_1_	AFB_2_	AFG_1_	AFG_2_	AFtotal	OTA
Mean	36.65	9.67	2.14	0.47	48.93	14.14
Std. Error of Mean	9.95	1.97	0.68	0.71	11.85	2.783
Median	16.21	7.62	0.00	0.00	25.09	6.70
Std. Deviation	54.50	10.83	3.72	0.93	64.92	15.25
Variance	2970.10	117.25	13.80	0.88	4214.84	232.47
Skewness	2.69	0.75	1.93	1.82	2.14	0.85
Std. Error of Skewness	0.43	0.43	0.43	0.43	0.43	0.43
Kurtosis	8.59	− 0.68	3.78	2.10	5.37	− 0.58
Std. Error of Kurtosis	0.83	0.83	0.83	0.83	0.83	0.83
Minimum	0.00	0.00	0.00	0.00	0.00	0.00
Maximum	255.50	34.73	15.00	3.23	285.31	46.77
t	3.68	4.89	3.15	2.76	4.13	5.08
Sig. (2-tailed)	0.001	0.000	0.004	0.010	0.000	0.000
Percentiles 25	0.00	0.00	0.00	0.00	0.00	1.20
50	16.21	7.62	0.00	0.00	25.09	6.70
75	43.24	19.20	4.25	0.27	67.83	25.18

**Table 6:** Associations of mycotoxins (AFB_1_, AFtotal and OTA) in maize from Northern Region.

	AFB_1_	AFtotal	OTA
AFB_1_	1.00	0.907**	0.569**
		0.00	0.000
AFtotal		1.00	0.632**
			0.00
OTA			1.000

**Correlation is significant at the 0.01 level (2-tailed).

**Table 7:** Statistical summary of data obtained from the Ashanti Region of Ghana.

N samples (30)	AFB_1_	AFB_2_	AFG_1_	AFG_2_	AFtotal	OTA
Mean	43.84	13.03	4.45	1.07	62.37	11.52
Std. Error of Mean	9.32	2.36	1.04	0.35	11.90	3.67
Median	27.51	12.47	0.00	0.00	46.77	0.00
Std. Deviation	51.10	12.94	5.69	1.90	65.18	20.10
Variance	2611.33	167.34	32.43	3.60	4247.89	403.94
Skewness	1.58	0.67	0.98	1.73	1.12	1.75
Std. Error of Skewness	0.43	0.43	0.43	0.43	0.43	0.43
Kurtosis	2.32	− 0.37	− 0.24	2.10	0.71	2.50
Std. Error of Kurtosis	0.83	0.83	0.83	0.83	0.83	0.83
t	4.70	5.52	4.28	3.08	5.24	3.14
Sig. (2-tailed)	0.000	0.000	0.000	0.005	0.000	0.004
Minimum	0.00	0.00	0.00	0.00	0.00	0.00
Maximum	188.87	45.84	18.63	6.73	230.00	76.05
Percentiles 25	1.42	0.00	0.00	0.00	1.42	0.00
50	27.50	12.47	0.00	0.00	46.77	0.00
75	62.17	22.23	10.20	2.16	95.93	25.98

**Table 8:** Associations of mycotoxins (AFB_1_, AFtotal and OTA) in maize from Ashanti Region.

	AFB_1_	AFtotal	OTA
AFB_1_	1.00	0.978**	0.816**
		0.00	0.00
AFtotal		1.00	0.828**
			0.00
OTA			1.000

**Correlation is significant at the 0.01 level (2-tailed).

**Table 9:** Statistical summary of data obtained from the Eastern Region of Ghana.

N samples (30)	AFB_1_	AFB_2_	AFG_1_	AFG_2_	AFtotal	OTA
Mean	45.14	14.22	1.93	0.55	53.14	17.75
Std. Error of Mean	14.79	5.23	0.94	0.27	19.00	5.25
Median	5.02	0.31	0.00	0.00	4.83	4.10
Std. Deviation	81.00	28.67	5.13	1.47	104.12	28.77
Variance	6560.97	822.10	26.33	2.16	10,840.01	827.54
Skewness	2.22	2.06	3.61	2.56	2.48	1.78
Std. Error of Skewness	0.43	0.43	0.43	0.43	0.43	0.43
Kurtosis	5.00	3.02	14.26	5.44	6.24	1.97
Std. Error of Kurtosis	0.83	0.83	0.83	0.83	0.83	0.83
t	3.05	2.72	2.06	2.05	2.80	3.38
Sig. (2-tailed)	0.005	0.011	0.049	0.050	0.009	0.002
Minimum	0.00	0.00	0.00	0.00	0.00	0.00
Maximum	337.30	101.00	24.80	5.51	441.02	97.51
Percentiles 25	0.00	0.00	0.00	0.00	0.00	0.00
50	5.02	0.31	0.00	0.00	4.83	4.10
75	47.19	9.40	1.07	0.00	47.60	20.31

**Table 10:** Associations of mycotoxins (AFB_1_, AFtotal and OTA) in maize from Eastern Region.

	AFB_1_	AFtotal	OTA
AFB_1_	1.00	0.909**	0.780**
		0.00	0.000
AFtotal		1.00	0.826**
			0.00
OTA			1.000

**Correlation is significant at the 0.01 level (2-tailed).

**Table 11:** Statistical summary of data obtained from the Central Region of Ghana.

N samples (30)	AFB_1_	AFB_2_	AFG_1_	AFG_2_	AFtotal	OTA
Mean	25.09	5.11	0.86	0.21	30.68	4.92
Std. Error of Mean	5.69	1.24	0.24	0.11	6.88	1.64
Median	15.30	3.66	0.00	0.00	20.79	0.00
Std. Deviation	31.15	6.79	1.30	0.57	37.66	8.99
Variance	970.58	46.09	1.69	0.33	1418.09	80.94
Skewness	1.43	2.73	1.27	2.49	1.54	0.43
Std. Error of Skewness	0.43	0.43	0.43	0.43	0.43	0.43
Kurtosis	0.94	9.93	0.28	4.82	1.65	6.07
Std. Error of Kurtosis	0.83	0.83	0.83	0.83	0.83	0.83
t	4.41	4.13	3.61	2.05	4.46	3.00
Sig. (2-tailed)	0.000	0.000	0.001	0.049	0.000	0.006
Minimum	0.00	0.00	0.00	0.00	0.00	0.00
Maximum	102.77	33.43	3.96	1.97	139.71	38.34
Percentiles 25	0.00	0.00	0.00	0.00	0.00	0.00
50	15.31	3.66	0.00	0.00	20.79	0.00
75	30.28	6.44	1.62	0.00	33.63	6.18

**Table 12:** Associations of mycotoxins (AFB_1_, AFtotal and OTA) in maize from Central Region.

	AFB_1_	AFtotal	OTA
AFB_1_	1.00	0.991**	0.780**
		0.00	0.000
AFtotal		1.00	0.814**
			0.000
OTA			1.00

**Correlation is significant at the 0.01 level (2-tailed).

**Table 13:** Statistical summary of data obtained from the Western Region of Ghana.

N samples (30)	AFB_1_	AFB_2_	AFG_1_	AFG_2_	AFtotal	OTA
Mean	43.86	12.64	4.55	1.07	62.12	24.19
Std. Error of Mean	8.94	1.88	0.94	0.33	10.88	4.44
Median	29.91	12.44	3.58	0.00	48.64	21.75
Std. Deviation	48.96	10.32	5.17	1.82	59.61	24.32
Variance	2396.60	106.42	26.73	3.30	3553.22	591.44
Skewness	1.73	0.26	0.69	1.58	1.16	0.93
Std. Error of Skewness	0.43	0.43	0.43	0.43	0.43	0.43
Kurtosis	3.02	− 1.10	− 1.01	1.69	0.99	0.19
Std. Error of Kurtosis	0.83	0.83	0.83	0.83	0.83	0.83
t	4.91	6.71	4.82	3.21	5.71	5.45
Sig. (2-tailed)	0.000	0.000	0.000	0.003	0.000	0.000
Minimum	0.00	0.00	0.00	0.00	0.00	0.00
Maximum	192.29	30.23	14.63	6.60	217.41	85.90
Percentiles 25	5.70	0.00	0.00	0.00	8.70	0.00
50	29.91	12.44	3.58	0.00	48.64	21.75
75	61.42	20.98	10.21	2.28	90.37	39.22

**Table 14:** Associations of mycotoxins (AFB_1_, AFtotal and OTA) in maize from Western Region.

	AFB_1_	AFtotal	OTA
AFB_1_	1.00	0.900**	0.655**
		0.00	0.000
AFtotal		1.00	0.680**
			0.000
OTA			1.00

**Correlation is significant at the 0.01 level (2-tailed).

The European Food Safety Authority (EFSA)^53^ and Ghana Standards Authority (GSA)^2,54^ regulatory limits for total aflatoxins (AFtotal) used were 4 and 10 µg/kg respectively. While ochratoxins (OTA) limits used were 4 and 5 µg/kg, respectively, for the two institutions^55^ (Table 15) in this study. Locally, both toxin quantity limits prescribed by the Ghana Standards Authority are a subset of the European Food Safety Authority (EFSA). Regarding the frequency and (percentage %) of positive (Yes) total aflatoxin (AFtotal) or ochratoxin A (OTA) contaminated maize samples above the various permissible limits, the Upper East region recorded an overall positive of 25/30, values of 25 (83.3%) and ranged between 7.00 and 106.18 µg/kg and 23 (76%) in range 10.76–106.18 µg/kg tested positive for EFSA and GSA_,_ respectively. For OTA, an overall positives of 20/30 were recorded. Values of 18 (60%) within the range of 4.88–62.50 µg/kg and 17 (56.67%) of the range 11.86–62.50 µg/kg tested positive for EFSA and GSA respectively. For Northern, 22/30 were positive with total aflatoxin values of 21 (70%) in the range 9.73–285.31 µg/kg. For OTA, 23/30 were positive with values of 18 (60%) which ranged between 4.70 and 46.77 µg/kg, and 17 (56.67%) of 3.30–46.77 µg/kg were recorded as positive for EFSA and GSA respectively. In the Ashanti Region, recorded 23/30 were positive with total aflatoxin values of 21 (70%) within the range of 7.98–230.00 µg/kg and 18 (60%) within the range of 19.40–230.00 µg/kg were recorded. OTA showed 10/30 positive, while 10 (33.33%) within the range of 5.80–76.05 µg/kg and 10 (33.33%) in range 5.80–76.05 µg/kg for EFSA and GSA. In the Eastern Region, 17/30 were positive and total aflatoxin values of 16 (53.3%) within a range of 4.72–441.02 µg/kg and 12 (40%) within a range of 10.70–441.02 µg/kg were recorded. OTA levels of 16/30 were positive and 15 (50%) with a range of 5.50–97.51 µg/kg were recorded for EFSA while 16 (53.33%) within a range of 5.50–97.51 µg/kg was recorded for GSA. The Central Region recorded 20/30 as positive, while total aflatoxin values of 20 (66.7%) within the range of 8.86–139.71 µg/kg and 19 (63.33%) within the range of 15.84–139.71 µg/kg were recorded. OTA showed 12/30 as positive while 11 (36.67%) within the range of 4.00–38.34 µg/kg and 8 (26.67%) in range 5.98–85.90 µg/kg for EFSA and GSA. Lastly, the Western Region recorded 24/30 positive samples and total aflatoxin values of 24 (80%) within the range of 4.27–217.41 µg/kg and 23 (76.67%) within the range of 10.18–217.41 µg/kg were recorded. OTA showed 22/30 positive and 22 (73.33%) within the range of 5.0–85.90 µg/kg and 21 (70.0%) in the range 5.0–85.90 µg/kg for EFSA and GSA respectively (Table 15). Out of a total of 180 samples analyzed for total aflatoxins (AFtotal), 127 (70.50%) surpassed the EFSA limit and were within the range of 4.27–441.02 µg/kg while 116 (64.44%) of the samples surpassed GSA limit and ranged between 10.18 and 441.02 µg/kg. Furthermore for OTA, 94 (52.22%) of the samples surpassed the tolerable limit of EFSA were within the range of 4.00–97.51 µg/kg, while 89 (49.44%) surpassed the limit of GSA and ranged between 3.30 and 97.51 µg/kg (Table 15).

**Table 15:** Proportions of samples that exceeded AFtotal and OTA and limits of the European Food Safety Authority (EFSA) and Ghana Standard Authority (GSA).

Region	No. of samples	Overall Positive	Exceeding EFSA regulation		Exceeding GSA regulation	
			Yes (%)	Range (µg/kg)	Yes (%)	Range (µg/kg)
**AFT**						
Upper	30	25	25 (83.3)	7.0–106.18	23 (76)	10.76–106.18
Northern	30	22	21 (70)	9.73–285.31	18 (60)	11.50–285.31
Ashanti	30	23	21 (70)	7.98–230.00	21 (70)	19.40–230.00
Eastern	30	17	16 (53.3)	4.72–441.02	12 (40)	10.70–441.02
Central	30	20	20 (66.7)	8.86–139.71	19 (63.33)	15.84–139.71
Western	30	24	24 (80)	4.27–217.41	23 (76.67)	10.18–217.41
*Total*	*180*	*131*	*127 (70.50)*	*4.27–441.02*	*116 (64.44)*	*10.18–441.02*
**OTA**						
Upper	30	20	18 (60.0)	4.88–62.50	17 (56.67)	11.86–62.50
Northern	30	23	18 (60.0)	4.70–46.77	17 (56.67)	3.30–46.77
Ashanti	30	10	10 (33.33)	5.80–76.05	10 (33.33)	5.8–76.05
Eastern	30	16	15 (50)	5.50–97.51	16 (53.33)	5.50–97.51
Central	30	12	11 (36.67)	4.00–38.34	8 (26.67)	5.98–38.34
Western	30	22	22 (73.33)	5.0–85.90	21 (70.0)	5.0–85.90
*Total*	*180*	*103*	*94 (52.22)*	*4.00–97.51*	*89 (49.44)*	*3.30–97.51*

Total values are in [italics].

European Union Food Safety (EFSA) limit for AF_Total_ = 4 µg/kg.

European Union Food Safety (EFSA) limit for OTA = 4 µg/kg.

Ghana Standards Authority (GSA) limit for AF_Total_ = 10 µg/kg.

Ghana Standards Authority (GSA) limit for OTA = 5 µg/k.

### Consumer risk assessment

#### Aflatoxins

The Estimated Daily Intakes (EDI) of total aflatoxins in the maize samples from the Upper East Region were 610, 180, 140, 80, and 60 ng/kg bw/day for infants, toddlers, children, adolescents, and adults respectively. The Margin of Exposure (MOE) values recorded were 0.66, 2.22, 2.86, 5.00, and 6.67, respectively. The average potency of the aflatoxins was 0.0323 aflatoxins ngkg^−1^ bwday^−1^ and produced cancer risks of 19.70, 5.81, 4.52, 2.58 and 1.94 cases/100,000 person/year respectively (Table 16). The Northern Region recorded EDI values of 900, 270, 200, 110, and 86 ng/kg bw/day for infants, toddlers, children, adolescents, and adults, respectively. MOE values of 0.40, 1.48, 2.00, 3.64, and 4.65. Cancer risks of 29.07, 8.72, 6.46, 3.55, and 2.78 cases/100,000 person/year respectively, for these age categories were recorded. For the Ashanti Region, the EDI values recorded for infants, toddlers, children, adolescents, and adults were 1150, 340, 260, 140, and 110 ng/kg bw/day respectively. MOE values recorded were 0.35, 1.18, 1.54, 2.86, and 3.64, respectively. The average potency was the same while the Cancer risks were 37.15, 10.98, 8.40, 4.52, and 3.55, cases/100,000 person/year respectively (Table 16). For the Eastern Region, the recorded EDI values recorded for infants, toddlers, children, adolescents, and adults were 980, 209, 220, 120, and 90 ng/kg bw/day, respectively. MOE values recorded were 0.41, 1.38, 1.82, 3.33 and 4.44, respectively. The average potency was the same while the Cancer risks were 31.65, 9.37, 7.11, 3.88, and 2.91 cases/100,000 person/year, respectively (Table 16). In the Central Region, the EDI values recorded for infants, toddlers, children, adolescents, and adults were 570, 170, 130, 70, and 50 ng/kg bw/day, respectively. MOE values recorded were 0.70, 2.35, 3.08, 5.71, and 8.00, respectively. The average potency was the same while the Cancer risks were 18.41, 5.49, 4.20, and 1.62, respectively (Table 16). Lastly, for the Western Region, the EDI values recorded for infants, toddlers, children, adolescents, and adults were 1150, 340, 260, 140, and 110 ng/kg bw/day respectively. MOE values recorded were 0.0107, 0.0307, and 0.0465, respectively. The average potency was the same while the Cancer risks were 37.15, 10.98, 8.40, 4.52, and 3.55 cases/100,000 person/year, respectively (Table 16).

**Table 16:** Evaluation of risk for Total Aflatoxins via consumption of maize.

Region	Age Category	Estimated Daily Intake (EDI) (ng/kg bw/day)	MOE	Av. Potency (ng Aflatoxins/kg bw/day)	Cancer Risk (Cases/100,000 persons/year)
Upper East	Infants (0–11 months)	610	0.66	0.0323	19.70
	Toddlers (12–35 months)	180	2.22	0.0323	5.81
	Children (36 months–10 years)	140	2.86	0.0323	4.52
	Adolescents (11–17 years)	80	5.00	0.0323	2.58
	Adults (18–64 years)	60	6.67	0.0323	1.94
Northern	Infants (0–11 months)	900	0.40	0.0323	29.07
	Toddlers (12–35 months)	270	1.48	0.0323	8.72
	Children (36 months–10 years)	200	2.00	0.0323	6.46
	Adolescents (11–17 years)	110	3.64	0.0323	3.55
	Adults (18–64 years)	86	4.65	0.0323	2.78
Ashanti	Infants (0–11 months)	1150	0.35	0.0323	37.15
	Toddlers (12–35 months)	340	1.18	0.0323	10.98
	Children (36 months–10 years)	260	1.54	0.0323	8.40
	Adolescents (11–17 years)	140	2.86	0.0323	4.52
	Adults (18–64 years)	110	3.64	0.0323	3.55
Eastern	Infants (0–11 months)	980	0.41	0.0323	31.65
	Toddler (12–35 months)	290	1.38	0.0323	9.37
	Children (36–10 years)	220	1.82	0.0323	7.11
	Adolescents (11–17 years)	120	3.33	0.0323	3.88
	Adults (18–64 years)	90	4.44	0.0323	2.91
Central	Infants (0–11 months)	570	0.70	0.0323	18.41
	Toddlers (12–35 months)	170	2.35	0.0323	5.49
	Children (36–10 years)	130	3.08	0.0323	4.20
	Adolescents (11–17 years)	70	5.71	0.0323	2.26
	Adults (18–64 years)	50	8.00	0.0323	1.62
Western	Infants (0–11 months)	1150	0.35	0.0323	37.15
	Toddlers (12–35 months)	340	1.18	0.0323	10.98
	Children (36–10 years )	260	1.54	0.0323	8.40
	Adolescents (11–17 years )	140	2.86	0.0323	4.52
	Adults (18–64 years )	110	3.64	0.0323	3.55

Margin of Exposure-MOE.

Mean aflatoxins- Upper East = 32.84 µg/kg, Brong = 48.93 µg/kg, Ashanti = 62.37 µg/kg, Eastern = 53.14 µg/kg.

Central = 30.68 µg/kg, Western = 62.12 µg/kg.

Daily intake of maize for infants and toddlers were halved (0.5 × 0.107 kg/day).

Daily intake of 0.107 kg/day was used for children, adolescents and adults.

Benchmark Dose Lower limit = 400 ng aflatoxins/kg bw/day.

#### Ochratoxins

Ochratoxins A (OTA) in the maize samples obtained from various regions produced varied results. In the Upper East Region, the Estimated Daily Intake (EDI) values recorded were 340, 100, 70, 40, and 30 ng/kg bw/day for infants, toddlers, children, adolescents, and adults, respectively. The Margin of Exposure (MOE) values recorded were 0.05, 0.18, 0.26, 0.45, and 0.60, respectively, for the same age categories. The average potency of the ochratoxins was 17.86 ochratoxins ng/kg bw/day which produced cancer risks of 10.98, 3.23, 2.26, 1.29, and 0.97 cases/100,000 person/year respectively (Table 17). The Northern Region recorded EDI values of 260, 80, 50, 30, and 30 ng/kg bw/day for infants, toddlers, children, adolescents, and adults, respectively. MOE values of 0.07, 0.22, 0.36, 0.60 and 0.60 were obtained respectively for the same age categories. Cancer risk values of 8.40, 2.58, 1.62, 0.97, and 0.97 cases/100,000 person/year respectively, were obtained. In the Ashanti Region, the EDI values recorded for infants, children, and adolescents and adults were 1150, 63, 50, 30, and 20 ng/kg bw/day, respectively. MOE values recorded were 0.35, 0.28, 0.36, 0.60, and 0.89. The average potency was the same for Upper East while the Cancer Risks were 37.15, 2.03, 1.62, 0.97, and 0.65 cases/100,000 person/year. (Table 17). In the Eastern Region, the EDI values recorded for infants, toddlers, children, adolescents, and adults were 330, 96, 70, 40, and 30 ng/kg bw/day, respectively. MOE values recorded were 0.05, 0.19, 0.26, 0.45, and 0.60, respectively. The average potency was the same while the Cancer risks were 10.66, 3.10, 2.26, 1.29, and 0.97 cases/100,000 person/year, respectively (Table 17). In the Central Region, the EDI values recorded for infants, toddlers, children, adolescents, and adults were 90, 30, 20, 10 and 8.6 × 10^–3^. Likewise, MOE values recorded were 0.20, 0.60, 0.89, 1.79, and 2059.97. The average potency was the same while Cancer Risks were 2.91, 0.97, 0.65, 0.32, and 2.78 × 10^–4^ cases/100,000 person/year (Table 17). Lastly, for the Western Region, the EDI values recorded for infants, toddlers, children, adolescents, and adults were 450, 130, 100, 60, and 43 ng/kg bw/day respectively. MOE values recorded were 0.04, 0.14, 0.18, 0.30, and 0.42, respectively. The average potency was the same as other regions, while the cancer risks were 14.54, 4.20, 3.23, 1.94, and 1.39 cases/100,000 person/year respectively (Table 17).

**Table 17:** Evaluation of risk for Ochratoxin A (OTA) via consumption of maize.

Region	Age Category	Estimated Daily Intake (EDI) (ng/kg bw/day)	MOE	Av. Potency (ng ochratoxins/kg bw/day)	Cancer Risk(Cases/100,000 persons/year)
Upper East	Infants (0–11 months)	340	0.05	0.0323	10.98
	Toddlers (12–35 months)	100	0.18	0.0323	3.23
	Children (36 months–10 years)	70	0.26	0.0323	2.26
	Adolescents (11–17 years)	40	0.45	0.0323	1.29
	Adults (18–64 years)	30	0.60	0.0323	0.97
Northern	Infants (0–11 months)	260	0.07	0.0323	8.40
	Toddlers (12–35 months)	80	0.22	0.0323	2.58
	Children (36–10 years )	50	0.36	0.0323	1.62
	Adolescents (11–17 years)	30	0.60	0.0323	0.97
	Adults (18–64 years)	30	0.60	0.0323	0.97
Ashanti	Infants (0–11 months)	210	0.09	0.0323	6.78
	Toddlers (12–35 months)	63	0.28	0.0323	2.03
	Children (36–10 years)	50	0.36	0.0323	1.62
	Adolescents (11–17 years)	30	0.60	0.0323	0.97
	Adults (18–64 years)	20	0.89	0.0323	0.65
Eastern	Infants (0–11 months)	330	0.05	0.0323	10.66
	Toddlers (12–35 months)	96	0.19	0.0323	3.10
	Children (36 months–10 years)	70	0.26	0.0323	2.26
	Adolescents (11–17 years)	40	0.45	0.0323	1.29
	Adults (18–64 years)	30	0.60	0.0323	0.97
Central	Infants (0–11 months)	90	0.20	0.0323	2.91
	Toddlers (12–35 months)	30	0.60	0.0323	0.97
	Children (36 months–10 years)	20	0.89	0.0323	0.65
	Adolescents (11–17 years)	10	1.79	0.0323	0.32
	Adults (18–64 years)	8.6 × 10^–3^	2059.97	0.0323	2.78 × 10^–4^
Western	Infants (0–11 months)	450	0.04	0.0323	14.54
	Toddlers (12–35 months)	130	0.14	0.0323	4.20
	Children (36–10 years)	100	0.18	0.0323	3.23
	Adolescents (11–17 years)	60	0.30	0.0323	1.94
	Adults (18–64 years)	43	0.42	0.0323	1.39

Margin of Exposure-MOE.

Mean ochratoxin A- Upper East = 18.22 µg/kg, Northern = 14.14 µg/kg, Ashanti = 11.52 µg/kg, Eastern = 17.75 µg/kg.

Central = 4.92 µg/kg, Western = 24.19 µg/kg.

Daily intake of maize for infants and toddlers were halved (0.5 × 0.107 kg/day).

Daily intake of 0.107 kg/day was used for children, adolescents, and adults.

Benchmark Dose Lower limit = 17.86 ng ochratoxins/kg bw/day.

## Discussion

The discrete incidence of ochratoxins and aflatoxins in foodstuffs is quite common in cereals and is a worldwide problem during pre-and post-harvest stages^56^. However, the concomitant occurrence of these mycotoxins has not been researched adequately in Africa. In this study, a range of 0–97.51 μg/kg (mean 48.76 μg/kg) of ochratoxins was obtained and were comparable to other previous studies globally. Nalle et al.^57^ reported a mean value of 20.385 μg/kg in corn grains and corn products from different sources in West Timor, Indonesia. Kamala et al.^58^ reported a range of 16–73 μg/kg OTA concentration in maize samples from Tanzania. Values of the ranges 0.12–0.59 μg/kg and 1.70–19.5 μg/kg have been reported by Kara et al.^59^ in cereal flour samples from Turkey and by De Giromalo et al.^60^ for cereals from Italy respectively, Again, Temba et al.^61^ also reported levels of 19.5 μg/kg. Veldman et al.^62^ demonstrated a total of 16.7% of maize samples originating from Holland contaminated by OTA with an average level of 73 μg/kg. From Brazil, Sekiyama et al.^63^ recorded levels between the range of 0–64 μg/kg. Greater quantities of ochratoxins have been reported in maize from other studies worldwide. Recently, ul Hassan et al. ^64^ reported ochratoxins (OTA) levels that ranged from 2.14 to 214 μg/kg which was detected in 71% of commercially grown maize samples in Pakistan. Makun et al.^65^ reported that samples of maize showed the highest levels of OTA were in the range of ND to 139.2 μg/kg in Nigeria. Conversely, Iram et al.^66^ did not detect ochratoxins in maize samples obtained from Punjab, Pakistan. Adebajo et al.^67^ also detected OTA in corn-based snacks in Nigeria only at toxicologically significant levels. The co-occurrence of different mycotoxins in one commodity created by fungal genera is common^68^. The permissible limit of OTA recognized by FAO/WHO Joint Committee Experts on Food Additives is 100 ng/kg/week and 14 ng/kg/day, whereas European Food Safety reestablished 120 ng/kg of OTA, which is nearly 17.1 ng/kg^47^. Ochratoxins have been implicated in a variety of adverse health effects both in humans and in animals suggested to be reached from renal, neuro-, immuno-, and embryo toxicity to muta- or teratogenicity^69^. OTA was proven as a renal carcinogen in rodents^70^, nevertheless its transferability to humans is still not clear^71^. Its carcinogenicity has been adopted to be related with genetic changes leading to a new assessment so that OTA can also be considered as genotoxic/mutagenic^72^. The high presence of OTA might be attributed to other fungal species that have not yet been explored or due to other pieces.

Aflatoxin level recorded in this study were in the range of 0–441.02 μg/kg, which is within the range of the values reported by Adebajo et al.^67^ for values between the ranges 25–770 ppb, 15–1070 ppb, and 10–160 ppb for corn, corn cake, and corn roll snack respectively in Nigeria. Recently, Kortei et al.^2^ also reported aflatoxins levels of 0–445.01 μg/kg from maize samples obtained from different agro-ecological zones across Ghana. Aflatoxin quantities of 692 and 945 ng/g from maize samples obtained from Fumesua and Ejura in Ghana respectively by^73^. From Eastern and Central Kenya, Lewis et al.^74^ reported greater quantities of aflatoxin > 1000 ppb in maize samples from contamination due to aflatoxin of commercial maize products during an outbreak of acute aflatoxicosis. Dadzie et al.^73^ recorded Total aflatoxins levels in the samples per community were in the range of limit of detection (LOD) with 692 ng/g, 23 ng/g, 945 ng/g, and 112 ng/g for Fumesua, Wenchi, Ejura, and Akomadan, respectively. Lower aflatoxin levels have been reported in other studies. de Souza et al.^75^ reported quantities as low as 0–16 in Brazilian maize and maize-based foods. Total aflatoxin values of 50.234, 70.102, and 30.943 ng/g were, respectively, obtained from three composite samples taken from the Ejura market was reported by^76^. Danso et al.^77^ reported aflatoxin levels of 2.9–3.4 ppb in all markets in the minor season maize samples, but levels ranging from 38.2 to 64.0 ppb were found in the major season samples. Total aflatoxin levels of 82.9 ppb, 48.9 ppb, and 48.9 ppb were recorded for maize samples stored in polypropylene sack, hermetic bags, and local crib respectively by^78^.

The variation in contamination levels as seen in the different agro-ecological zones could be attributed to speckled infection levels of the toxigenic fungus genus, *Aspergillus (esp. flavus),* owing to their ubiquitous nature*,* infect maize grains in the field even before harvest^79,80^. Ghana is reliant on rain-fed agriculture which is coupled with high temperatures and unavailability of regular rains, the crop is left under stress which predisposes the crop to fungal invasion^81^ during the growth cycle. This may explain the comparatively greater quantities of aflatoxins on maize obtained from the Easter Region since the region is endowed with sufficient rainfall and relatively high temperatures which induce aflatoxin production. Additionally, the different water activity levels of the grains due to the drying process used before and during storage could account for the different mycotoxin levels.

There were significant correlations between AFtotal and OTA. Likewise, AFB_1_ and AFtotal in this study. These observations corroborate the findings of some previous researchers^63,64,66,82^. The co-existence of two or more fungi and their subsequent mycotoxin production in an environment suggests a possible non-antagonistic metabolite interaction, Moreover, the probable effect of combined exposure to aflatoxins with other mycotoxins in foodstuffs could be additive or antagonistic. At variance with our observation, Imperato et al.^82^ reported that among food products analyzed in Italy, dried vine fruits were mainly contaminated with OTA and less with AFs. Discovered from the pertinent literature, there is an inverse correlation between AFB_1_ and OTA. To buttress this claim^83^ observed, no AFB_1_ was found in dried vine fruits, while OTA was detected at high levels. In addition, Dimitrokallis et al.^84^ reported that OTA inhibits AFB_1_ production by *Aspergillus species* in a related study.

The different categorizations of the biosphere (agro-ecological zones) presage different growth conditions for the proliferation of fungi. Brzonkalik et al.^85^ as well Garcia et al.^86^ emphasized mycotoxin production depends on species or/and strain, which is affected by the growth substrate and environmental conditions. de Souza et al.^75^ and Kortei et al.^88^ also explained co-existence of fungal strains on a substrate can affect both the level of mycotoxin production and the toxicity of the contaminated grains resulting in additive and synergistic effects when tested Nonetheless, data on multiple mixtures is very rare^87^. In this study the relatively high aflatoxin (AF) and ochratoxin (OTA) concentrations in maize grains obtained in the Eastern Region is expected as it geographically falls in a semi-deciduous zone with mean rainfall and temperatures of 1400–1900 mm and 25.9 °C respectively suggesting favorable environmental conditions for the growth of these toxigenic fungi^88,89^ implicated. Pitt and Hocking^90^ noted that *Penicillium* spp. and *Aspergillus spp*. grow well between 0 and 30 °C and have been found to produce greater quantities of ochratoxin A and aflatoxins respectively, this may explain the high incidence of this mycotoxin. Furthermore, Magan et al.^89^ also emphasized fungal contamination in cereals is influenced by two main factors. Firstly, by initial high moisture content in crops or late harvesting of crops in rural areas, and secondly, the lack of poor storage facilities characterized by poor ventilation, high temperatures, and humidity.

Regular monitoring and pre-and post-harvest control measures can be used to control mycotoxins by enhancing the resistance of the crop to incursive fungi through plant breeding or genetic engineering, which are laborious and time-consuming. Effective, sustainable, and universally applicable preharvest intervention strategies are needed. Proper field management practices such as the use of resistant varieties, timely planting, fertilizer application, weed control, insect control, and avoiding drought and nutritional stress could reduce mycotoxins^91^. Other options are biological control by using non-toxigenic strains to competitively displace toxigenic fungi^92^.

## Risk assessment

Kuiper-Goodman^93^ highlighted that risk estimations are computed to envisage the adverse health implications of mycotoxin exposure and guide food regulators to set limits for these toxins in foodstuffs. Risk assessment results obtained in this study were comparable to the published findings of Blanckson and Mills-Robertson^94^ as they reported Total aflatoxins EDI values of 0.005–1.054 μg/kg bw/day and 0.004–0.838 μg/kg bw/day for infants and young children respectively in infant cereal-based formula and were risky for children to consume in Ghana. The Estimated Daily Intakes (EDI) of total aflatoxins in the maize samples recorded by Kortei et al.^2^ were 109.7, 58.8, 33.08, and 25.2 μg/kg bw/day for infants, children, adolescents, and adults with Margin of Exposure (MOE) values of 4.73, 8.79, 15.63 and 20.51 respectively. Exposure assessment carried out by Omari and Anyebuno^95^ showed that the minimum and maximum daily AFS exposures were 0.044 and 2.805 µg/kg bw/day, respectively for weanimix (infant complementary food prepared from maize) for rural households; these rates for complementary food purchased from urban shops were 0.014 and 0.55 µg/kg bw/day, respectively. The chances of liver cancer development would increase to 0.6 per year if infants were fed with complementary food prepared in rural households with a minimum AF level of 7.9 µg/kg.

In Guangzhou China, Zhang et al.^96^ reported EDI values of range 0.02–0.04 respectively, for the age ranges of 3–6, 7–17, 18–59, and above 60 years for maize and products. All their computed MOE values were below the safe threshold of 10,000 and so the risk analysis results showed that most of the lower bound MOE values ranged from 10 to 100, indicating a concern for risk management. Age-group analysis suggested close attention were paid to the 3 ~ 6 years of age group, whose MOE value was the lowest. Their results reflected that preschool children might have the highest risk of being exposed to AF. Their results agreed with our findings. The MOE values (995–860 at mean and 336 at 95th percentile exposure) and cancer potency estimates, based on the current exposure levels indicated a potential health concern for Turkish adults was reported by^97^. Li et al.^98^ pointed from a Chinese survey data, that the average daily intake of AFB_1_ from maize in the high-risk area was 184.1 µg, and the probable daily intake is estimated to be 3.68 µg/kg bw/day. Chun et al.^99^ estimated excess cancer risk values for liver cancer incidence by ingestion of these foods for AFB_1_ were calculated to be 5.78 × 10^–6^ for individuals negative for hepatitis B and 1.48 × 10^–4^ mg/kg bw/day for individuals positive for hepatitis B in Korea.

In this study, EDI were slightly greater compared with other EDI values reported globally. This implies a significant impact of aflatoxins on the nutritional status of humans and animals. Omari et al.^54^ observed that aflatoxin exposures were to some extent linked to nutrient deficiency in humans following a suggestion that aflatoxin exposure expedites intestinal damage resulting in a decline in nutrient absorption.

A strong association between anaemia and aflatoxin has been reported in Ghana showing that aflatoxin exposure may contribute partly to the high iron deficiency prevalent in children in developing countries including Ghana. The consumption of aflatoxins at high levels in a single dose or repeatedly for a brief period induces acute intoxication, henceforward labeled aflatoxicosis, in humans and animals with typical symptoms, including jaundice, lethargy, nausea, edema, hemorrhagic necrosis of liver tissues, bile duct hyperplasia, and eventually death (10–60%) subsequent to severe liver damage^100^. Although there is no consensus on the specific dose of aflatoxins that triggers acute toxicity in humans, it is well established that such a dose is highly variable depending on many factors, including the age, gender, health and nutritional status, presence or absence of underlying factors (e.g., chronic viral hepatitis, alcoholism, smoking, cirrhosis, exposure to hepatotoxic microcystins); and it is lowest in youngsters, as substantiated by the highest death rates of this age-group in aflatoxicosis outbreaks. A scoping review by Wu^100^ have shown the presence of these aflatoxins appeared in greater proportion in kwashiorkor of children and in different organs (brain, heart, kidney, liver, and lung) and biological samples (serum, stool, urine). This observation is particularly worrying since the Eastern Region of Ghana, is a major crop producing zone where most of the foodstuffs (maize, groundnuts etc.) used as ingredients for the production of local weaning formulas for humans and then animal feeds for poultry and livestock are produced due to favorable climatic conditions.

Various interventions have been established to combat aflatoxin biosynthesis and accumulation, ranging from preharvest to dietary interventions. Simply avoiding or reducing the consumption of foods that are frequently contaminated with aflatoxin has shown effectiveness in reducing liver cancer mortality in one population^100^. Advocacy on strict compliance with good agricultural practice (GAP), good manufacturing practice (GMP), as well as good hygiene practice (GHP), which are critical ingredients to alleviate the formation of aflatoxins in the field as well as during storage of foodstuffs, must be strengthened. By impeding aflatoxins formation in foods, there is the protection of both public health and the prevention of economic losses. Monitoring foods prone to fungal infection and the presence of mycotoxins regularly is cautious to assess the public level of awareness.

## Conclusion

Based on the results obtained in this study, per the permissible limits set by the European Food Safety Authority (EFSA) (2 and 4 µg/kg) and the Ghana Standards Authority (GSA) (5 and 10–15 µg/kg), it can be construed that out of the 180 samples analyzed for total aflatoxins (AFtotal), 68% exceeded the limits of EFSA whereas 58% exceeded GSA limits. In the case of AFB_1_, 71% of the samples exceeded EFSA limits while 64% exceeded GSA limits. For OTA, 94 (52.22%) of samples exceeded the tolerable limit of EFSA, while 89 (49.44%) exceeded the limits of GSA.

Health risk assessment for ochratoxin A as well as aflatoxin exposure via maize consumed in different regions of Ghana by infants, children, and adolescents, and adults showed a significant adverse health risk in all age categories of humans since all calculated values for MOE were less than 10,000 for both aflatoxins and ochratoxins. This study scratches the surface of a dire situation that calls for attention by all stakeholders involved in mitigating the harmful effects associated with these hazardous mycotoxin exposures to humans.

Presently, the prime challenge of mycotoxins proliferation in our foods is the link with climate change. There is now a widespread consensus that the world is warming at an unparalleled rate and this is expected to seriously affect our crop production as well as the phyllosphere microflora of these crops. The irrepressible growth of *A. flavus* under extreme heat and dry condition is an expected and emerging dilemma mainly in many parts of the world (Serbia, Hungary etc.) where there were very low reports of mycotoxin contamination. However, surges in maize contamination were observed after prolonged hot and dry weather. Due to this, the world’s largest agricultural food exporters such as Brazil, Argentina and some parts of Asia to include China and India have been identified as hot spots for impacts of climate change. From a food security viewpoint, a more precise forecast of impacts of climate change on mycotoxins need to be addressed to prevent conceded food sustainability which possibly results in negative social consequences.

Notwithstanding, some possible routes to improve the situation is the utilization of enzymatic biotransformation (purified enzymes) to degrade mycotoxins with much better precision in feeds/foods. Again the inclusion of binding agents or enterosorbents in the diet of humans or animals has been given considerable attention as a strategy to reduce foodborne exposures to mycotoxins by decreasing the mycotoxin availability thereby reducing its absorption. Recently, materials such as mycosorb (a new product which acts by binding pathogens and mycotoxins without affecting gut bacteria) and aluminosilicates have been reported to be adequately efficient in this regard. Furthermore, the use of biofuels and fermentation by-products such as distillers dried grains (DDGs) are some promising ways to drastically reduce the impacts of these mycotoxins on the gut and health of livestock and poultry. Lastly, a more practical means of curbing mycotoxicity is the diversification of our diets which include the non-adherence to a few food classes (esp. cereals and legumes) but an expanded one to include different classes is a more promising approach.

## Data Availability

All data supporting this study are included in the article and its supplementary material.
